# Exploiting natural variation in crown root traits via genome-wide association studies in maize

**DOI:** 10.1186/s12870-021-03127-x

**Published:** 2021-07-23

**Authors:** Houmiao Wang, Xiao Tang, Xiaoyi Yang, Yingying Fan, Yang Xu, Pengcheng Li, Chenwu Xu, Zefeng Yang

**Affiliations:** 1 Jiangsu Key Laboratory of Crop Genetics and Physiology/ Key Laboratory of Plant Functional Genomics of the Ministry of Education/ Jiangsu Key Laboratory of Crop Genomics and Molecular Breeding, Yangzhou, 225009 China; 2 Jiangsu Co-Innovation Center for Modern Production Technology of Grain Crops, Yangzhou University, Yangzhou, 225009 China; 3 Joint International Research Laboratory of Agriculture and Agri-Product Safety of Ministry of Education of China, Yangzhou University, Yangzhou, 225009 China

**Keywords:** Crown root traits, Maize, GWAS, Candidate genes, Natural variation

## Abstract

**Background:**

Root system architecture (RSA), which is determined by the crown root angle (CRA), crown root diameter (CRD), and crown root number (CRN), is an important factor affecting the ability of plants to obtain nutrients and water from the soil. However, the genetic mechanisms regulating crown root traits in the field remain unclear.

**Methods:**

In this study, the CRA, CRD, and CRN of 316 diverse maize inbred lines were analysed in three field trials. Substantial phenotypic variations were observed for the three crown root traits in all environments. A genome-wide association study was conducted using two single-locus methods (GLM and MLM) and three multi-locus methods (FarmCPU, FASTmrMLM, and FASTmrEMMA) with 140,421 SNP.

**Results:**

A total of 38 QTL including 126 SNPs were detected for CRA, CRD, and CRN. Additionally, 113 candidate genes within 50 kb of the significant SNPs were identified. Combining the gene annotation information and the expression profiles, 3 genes including GRMZM2G141205 (IAA), GRMZM2G138511 (HSP) and GRMZM2G175910 (cytokinin-O-glucosyltransferase) were selected as potentially candidate genes related to crown root development. Moreover, GRMZM2G141205, encoding an AUX/IAA transcriptional regulator, was resequenced in all tested lines. Five variants were identified as significantly associated with CRN in different environments. Four haplotypes were detected based on these significant variants, and Hap1 has more CRN.

**Conclusions:**

These findings may be useful for clarifying the genetic basis of maize root system architecture. Furthermore, the identified candidate genes and variants may be relevant for breeding new maize varieties with root traits suitable for diverse environmental conditions.

**Supplementary Information:**

The online version contains supplementary material available at 10.1186/s12870-021-03127-x.

## Background

The global human population is expected to reach 10 billion within the next 30 years[1]. Current yield increases for major crops will likely not be able to satisfy the future demand. Improving plant architecture, both above and below ground, is one of the most effective ways to increase crop productivity. During the Green Revolution, the manipulation of above-ground plant features resulted in substantial increases in cereal yields [2]. The roots, which serve as the interface between the plant and the dynamic soil environment, have crucial functions affecting plant productivity and tolerance to environmental stresses [3]. Research regarding plant roots has been limited by the complexity of phenotyping the underground plant parts and because there is relatively little relevant genetic information available to breeders. However, a recent study confirmed that modifying the root architecture can increase resource use efficiency and yields [4], which has prompted plant scientists to focus more of their attention on plant roots. Clarifying the genetic mechanism regulating root development is critical for enhancing crop performance and increasing food security.

Root system architecture (RSA) is the spatial deployment of roots. Crown roots form the majority of the maize root system’s backbone [5]. To characterise the effects of RSA on crop performance, several phenotyping methods involving artificial conditions (e.g., on germination paper) and natural conditions (e.g., field trials) have been developed to evaluate genotypic variations and the utility of root traits [6]. Moreover, shovelomics-based experiments have been widely used to investigate soil resource acquisition by maize crown roots [7]. Furthermore, maize crown root traits, including crown root number [8], diameter [9], and growth angle [4], are associated with the above-ground performance of plants as well as the nutrient content and grain yield. Decreasing the number of roots in maize inbred lines may lead to deeper root growth and enhanced acquisition of water and nitrogen in dry and low-nitrogen soils [8, 10]. In low crown root number phenotypes, roots may be too spatially dispersed to sufficiently acquire soil resources, low crown root number supports a deep root system, so the shallow portion of deep roots can acquire shallow N resources while the deep portion can explore deep soil for water resources [10]. Since water and nitrate enter deeper soil strata over time, root systems with rapid exploitation of deep soil would optimize water and N capture in most maize production environments [11]. The root angle is a crucial factor influencing how deep roots can grow in the soil to obtain water and nutrients. RSA that are narrow and vertically oriented are typically associated with increased drought tolerance [12]. In maize, rice and wheat, root growth depth is positively associated with yield under drought conditions[4, 13, 14]. Relatively steep root angles increase rooting depth and drought tolerance in rice [4] and common bean [15]. The root angle also affects nitrogen capture, with steep maize root angles under low-nitrogen conditions potentially increasing nitrogen acquisition by 15%–50% as the soil nitrogen content decreases [16, 17]. Compared with thin roots, thick roots have greater mechanical strength, leading to better anchorage and lodging resistance, protection against herbivory, and enhanced soil penetration [9]. Inbred lines exhibiting high nitrogen use efficiency (NUE) reportedly have larger root diameters than lines with a lower NUE under low-nitrogen conditions, and most genotypes decreased root diameter under stress when averaged across nodes [9]. An exposure to nitrogen stress induces several maize genotypes to increase their root diameter. Thus, the root diameter can influence adaptive stress responses. Accordingly, breeding maize lines with modified crown roots may lead to the development of new cultivars suitable for various stress conditions.

Exploring the natural variations in crown root traits may lead to new insights into root development, while also revealing elite allelic variations that can enhance root performance. Many recent quantitative trait locus (QTL) mapping studies regarding the number of maize crown roots have been conducted [18]. For example, QTL analyses in single and multiple environments have been performed for the crown root angle (CRA), the crown root diameter (CRD), and the crown root number (CRN) in a recombinant inbred line population. A total of 46 QTL were detected in a single environment and 25 QTL were identified in multiple environments, with most loci confirmed as minor-effect QTL for crown root traits [19]. Two major QTL for the total brace root number were identified by Ku et al. [20], and the largest additive effect was 16.4%–17.9%. Cai et al. detected five QTL for CRN at three maize growth stages, including one consensus QTL in chromosome bin 10.4 [21]. There have been relatively few studies on the maize CRA. Only 10 root angle-related QTL were identified in two maize–teosinte F_2_ populations [22]. In rice, six major QTL for root angle (*DRO1*, *DRO2*, *DRO3*, *DRO4*, *DRO5*, and *qSOR1*) have been identified, and *DRO1* and *qSOR1* have been cloned [23, 24]. Additionally, *DRO1* is the first cloned gene associated with the root growth angle. Its expression is negatively regulated by auxin and the encoded protein helps mediate root tip cell elongations related to asymmetrical root growth and the downward bending of the root in response to gravity [4]. A previous study confirmed that *qSOR1*, which is a *DRO1* homologue, is also negatively regulated by auxin, and is predominantly expressed in root columella cells to influence root gravitropic responses [24]. All of these genes are potential targets for root system architecture (RSA)-related breeding. Regarding the root diameter, only one QTL associated with CRD has been detected under well-watered and water stress conditions [25]. Moreover, 21 root architecture traits were evaluated in three recombinant inbred populations, but only one root diameter-related QTL was identified [26]. Six QTL were identified for four root anatomical traits which is directly related to root diameter [27].

To date, only a few maize studies have identified QTL and allelic variations associated with RSA development in the field. As an alternative to traditional QTL mapping and map-based cloning, a genome-wide association study (GWAS) can identify the genes and allelic variations responsible for natural phenotypic diversity. In this study, the CRN, CRA, and CRD of 316 maize inbred lines were evaluated in three field trials. The objectives of this study were (i) to study phenotypic variation of crown root traits within a maize association panel, (ii) to identify significant SNPs associated with CRA, CRD and CRN, and (iii) to detected potential candidate genes and natural variations for crown root development.

## Results

### Phenotypic variations

**Fig. 1:**
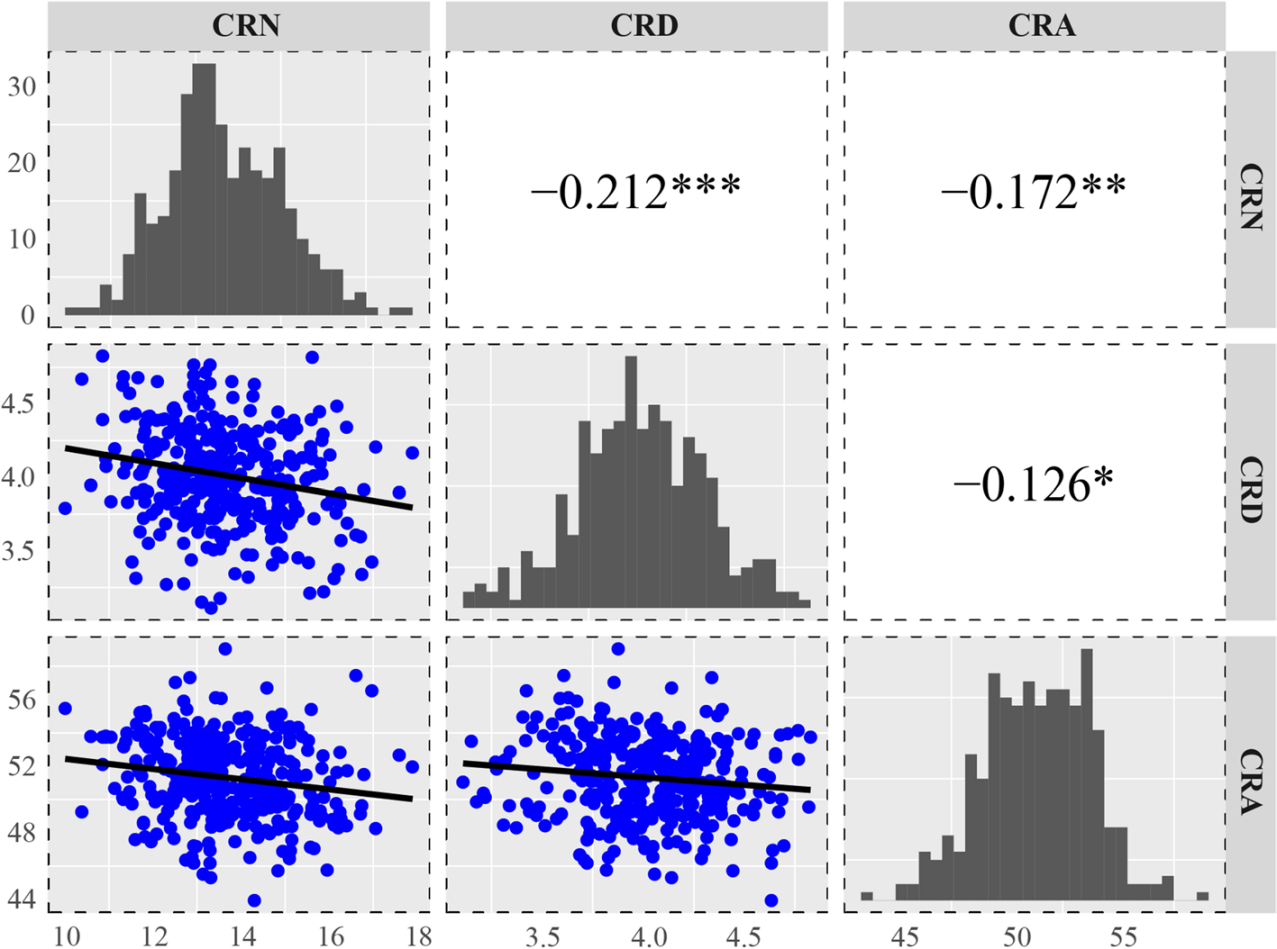
Phenotypic distribution and correlation of crown root angle (CRA), diameter (CRD) and number (CRN). ***: Significant correlation at *P* < 0.001

A total of 316 inbred lines from a diverse maize panel were evaluated regarding their CRA, CRD, and CRN at maturity in field trials across three environments. Phenotypic analyses confirmed the three crown root traits differed substantially in the examined environments (Table 1, Fig. 1). The coefficient of variation ranged from 4.72% to 9.87% for the BLUP values for the three environments. The frequency distributions of the analyzed crown root traits revealed a relatively normal distribution in each environment (Fig. 1). A two-way ANOVA indicated the effects of the genotype, the environment, and the genotype × environment interaction was significant for the three root traits (Table 1). On the basis of a correlation analysis of the crown root traits, a weak negative correlation between CRA, CRD and CRN was observed (Fig. 1).

**Table 1:** Descriptive statistics, ANOVA for crown root traits in different environments

Trait	Env	Min	Max	Mean ± SD	CV(%)	Heritability(%)	ANOVA^a^		
							**Env**	**G**	**Env × G**
CRA	2015SY	23.6	60.4	45.8 ± 6.12	13.37%	64.1	492.60**	3.65**	2.90**
	2016SY	30.0	69.5	53.8 ± 4.46	8.29%				
	2017SY	23.0	76.0	49.9 ± 7.71	15.42%				
	BLUP	43.9	59.0	51.2 ± 2.42	4.72%				
CRD	2015SY	2.9	5.7	4.2 ± 0.47	11.16%	64.4	174.42**	2.12**	1.24**
	2016SY	1.7	6.3	3.7 ± 0.65	17.60%				
	2017SY	2.0	7.0	4.1 ± 0.82	20.00%				
	BLUP	3.1	4.8	4.0 ± 0.32	8.17%				
CRN	2015SY	8.5	22.0	14.3 ± 2.10	14.69%	71.7	102.51**	2.91**	1.47**
	2016SY	7.0	25.0	12.8 ± 2.43	19.04%				
	2017SY	7.0	22.0	14.0 ± 2.59	18.45%				
	BLUP	10.4	17.9	13.7 ± 1.35	9.87%				

^a^the number indicated *F* value by Two-way ANOVA, and a significant effect by *F* test was indicated by “**” (*P* < 0.01)

### Genome-wide association study of crown root traits

**Fig. 2:**
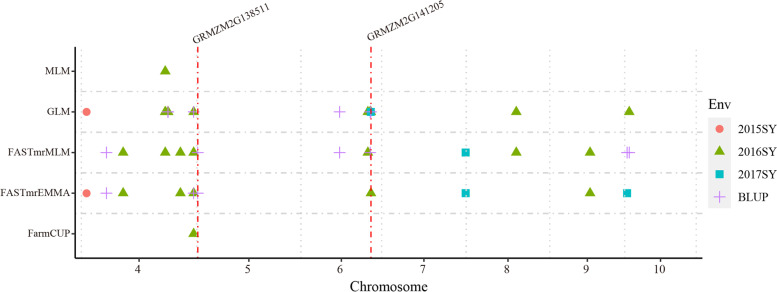
GWAS results for crown root number by three GWAS methods in different environments. The grouped region that was detected in at least two environments or by at least two models was displayed in this figure

To clarify the genetic basis of crown root traits, association analyses involving 140,421 SNP markers for the CRA, CRD, and CRN of the 316 genotypes were conducted using the following two single-locus methods: GLM and MLM, and three multi-locus methods: FarmCPU, FASTmrMLM, and FASTmrEMMA. A total of 482 marker–trait associations were identified (Table 2, Fig. 2, Table S1). More specifically, FASTmrMLM identified the most significant SNPs (216), followed by GLM (174), FASTmrEMMA (61), FarmCPU (33) and MLM (4). Additionally, 185, 127, and 170 significant SNPs were detected for CRA, CRD, and CRN, respectively, by all different methods. A total of 48, 209, and 59 significant SNPs were identified in 2015SY, 2016SY, and 2017SY, respectively, whereas 166 significant SNPs were detected based on the BLUP value for the three environments. Twenty-nine SNPs were identified by at least two methods, among these 17 loci were identified by single locus and multi-locus methods simultaneously (Table S1). Such as, SNP_5_158203765 and SNP_4_235330942, were detected by four GWAS methods except MLM. After clumped, a total of 38 QTL including 126 SNPs were detected for three root traits. Q7 including 7 significant SNP were detected in 2016SY and BLUP value for CRA. Q8 were detected in 2016SY and BLUP value for CRA by both GLM and MLM. Q17 associated with CRD could detected by GLM, FASTmrEMMA and FASTmrMLM. SNP_4_235330942 in Q29 were identified by GLM, FarmCPU, FASTmrEMMA and FASTmrMLM in 2016SY and BLUP value for CRN. Q33 including 10 significant SNP were detected by GLM, FASTmrEMMA and FASTmrMLM in 2016SY, 2017SY and BLUP value for CRN (Table 3). We compared the positions of the QTL identified in this study with the positions of the QTL reported from previous biparental studies [19]. Of the 38 loci we detected, three overlapped with QTL previously identified from bi-parental mapping studies (Table 3). Q19 associated with CRD was located within _*15*_*CRD6* and _*av*_*CRD6* identified by a previous QTL mapping. Another QTL associated with CRD, Q21, was located within confidence interval of _*av*_*CRD8*. A CRN QTL-Q23 was located within confidence interval of _*17*_*CRD4*.

**Table 2:** Summary of significant SNPs detected by different GWAS methods for crown root traits

Env	Trait	Single-locus methods		Multi-locus methods		
		**GLM**	**MLM**	**FASTmrMLM**	**FASTmrEMMA**	**FarmCPU**
2015SY	CRA	6	0	6	1	1
	CRD	1	0	10	6	0
	CRN	1	0	11	5	0
2016SY	CRA	55	2	21	6	6
	CRD	8	0	23	6	10
	CRN	27	1	26	8	10
2017SY	CRA	0	0	13	3	0
	CRD	1	0	14	2	0
	CRN	2	0	19	5	0
BLUP	CRA	40	0	18	7	0
	CRD	10	1	24	5	6
	CRN	23	0	25	7	0
Total		174	4	210	61	33

**Table 3:** Significant SNP for crown root traits detected by at least two models

Trait	QTL	ENV	SNP	Method	LOD	Previous QTL
CRA	Q1	2016SY	SNP_1_56897538	mrEMMA; mrMLM	6.96	
	Q2	2015SY	SNP_1_285593212	mrEMMA; mrMLM	4.58	
	Q3	2016SY	SNP_1_292661954	MrEMMA; mrMLM; GLM	5.49	
	Q4	2015SY	SNP_4_26119510	mrMLM; GLM	5.19	
	Q5	2017SY; BLUP	SNP_4_229202306	mrMLM	5.19	
	Q6	BLUP	SNP_5_32046071	mrEMMA; mrMLM	4.96	
	Q7	2016SY; BLUP	SNP_7_16560558	GLM	6.21	
	Q8	2016SY; BLUP	SNP_7_99321370	MLM; GLM	8.08	
	Q9	BLUP	SNP_7_103362548	mrMLM; GLM	5.68	
	Q10	2016SY; BLUP	SNP_7_103953346	mrEMMA; GLM	5.24	
	Q11	BLUP	SNP_7_104570406	mrEMMA; mrMLM; GLM	6.61	
	Q12	2017SY; BLUP	SNP_10_117747965	mrMLM	6.24	
CRD	Q13	2015SY	SNP_1_177406418	mrEMMA; mrMLM	4.92	
	Q14	BLUP	SNP_1_227741904	mrEMMA; mrMLM	6.49	
	Q15	2016SY; BLUP	SNP_3_191323638	GLM; FarmCPU	7.33	
	Q16	2016SY; BLUP	SNP_5_156094190	GLM	6.15	
	Q17	2016SY; BLUP	SNP_5_158203765	mrEMMA; mrMLM; GLM	18.24	
	Q18	2015SY; BLUP	SNP_5_166452989	mrEMMA; GLM	5.86	
	Q19	2016SY	SNP_6_112539707	mrEMMA; mrMLM; GLM	11.19	_*15*_*CRD6*; _*av*_*CRD6*
	Q20	2017SY; BLUP	SNP_7_173609433	mrMLM	4.12	
	Q21	2016SY; 2017SY	SNP_8_28739061	mrMLM	7.14	_*av*_*CRD8*
	Q22	2016SY; BLUP	SNP_10_125502748	mrMLM	5.93	
CRN	Q23	2015SY	SNP_4_10804837	mrEMMA; GLM	5.28	_*17*_*CRN4*
	Q24	BLUP	SNP_4_52541037	mrEMMA; mrMLM	4.41	
	Q25	2016SY	SNP_4_87583019	mrEMMA; mrMLM	5.61	
	Q26	2016SY	SNP_4_175786824	mrMLM; MLM; GLM	6.46	
	Q27	2016SY; BLUP	SNP_4_181565381	GLM	5.84	
	Q28	2016SY	SNP_4_207569283	mrEMMA; mrMLM	6.14	
	Q29	2016SY; BLUP	SNP_4_235330942	mrEMMA; mrMLM; GLM; FarmCPU	9.11	
	Q30	BLUP	SNP_5_1829751	mrEMMA; mrMLM	6.65	
	Q31	BLUP	SNP_6_81133730	mrMLM; GLM	5.71	
	Q32	2016SY	SNP_6_140298004	mrMLM; GLM	6.35	
	Q33	2016SY; 2017SY; BLUP	SNP_6_145084885	mrEMMA; mrMLM; GLM	7.31	
	Q34	2017SY	SNP_7_175781987	mrEMMA; mrMLM	6.20	
	Q35	2016SY	SNP_8_104966220	mrMLM; GLM	8.75	
	Q36	2016SY	SNP_9_84205355	mrEMMA; mrMLM	6.63	
	Q37	2017SY; BLUP	SNP_10_4729497	mrEMMA; mrMLM	5.09	
	Q38	2016SY; BLUP	SNP_10_9143425	mrMLM; GLM	5.31	

### Determination of candidate genes

A total of 113 potential candidate genes within 50 kb of the 126 significant SNPs were identified based on the GWAS results and the filtered predicted gene set from the annotated B73 reference maize genome (version 5b.60) (Table S2). To examine the expression profiles of these candidate genes, we compared the gene expression levels in different B73 maize tissues. The 25 genes that were not expressed in the crown roots (i.e., FPKM = 0) were eliminated and 88 genes were obtained. After combining the gene annotation information and the expression profiles, we selected 3 candidate genes potentially related to crown root development (Table S2). GRMZM2G141205, which encodes an Aux/IAA-transcription factor, was related with a CRN QTL-Q33. This gene was exclusively expressed in the crown roots. GRMZM2G175910 encoded a cytokinin-O-glucosyltransferase was around SNP_7_103946580 in Q10, which was associated with CRA. This gene was mainly expressed in the roots, stem, internode, cob and seed (Table S2). GRMZM2G138511 encoded a heat shock protein, which was associated with CRN.

### Resequencing and a haplotype analysis

**Fig. 3:**
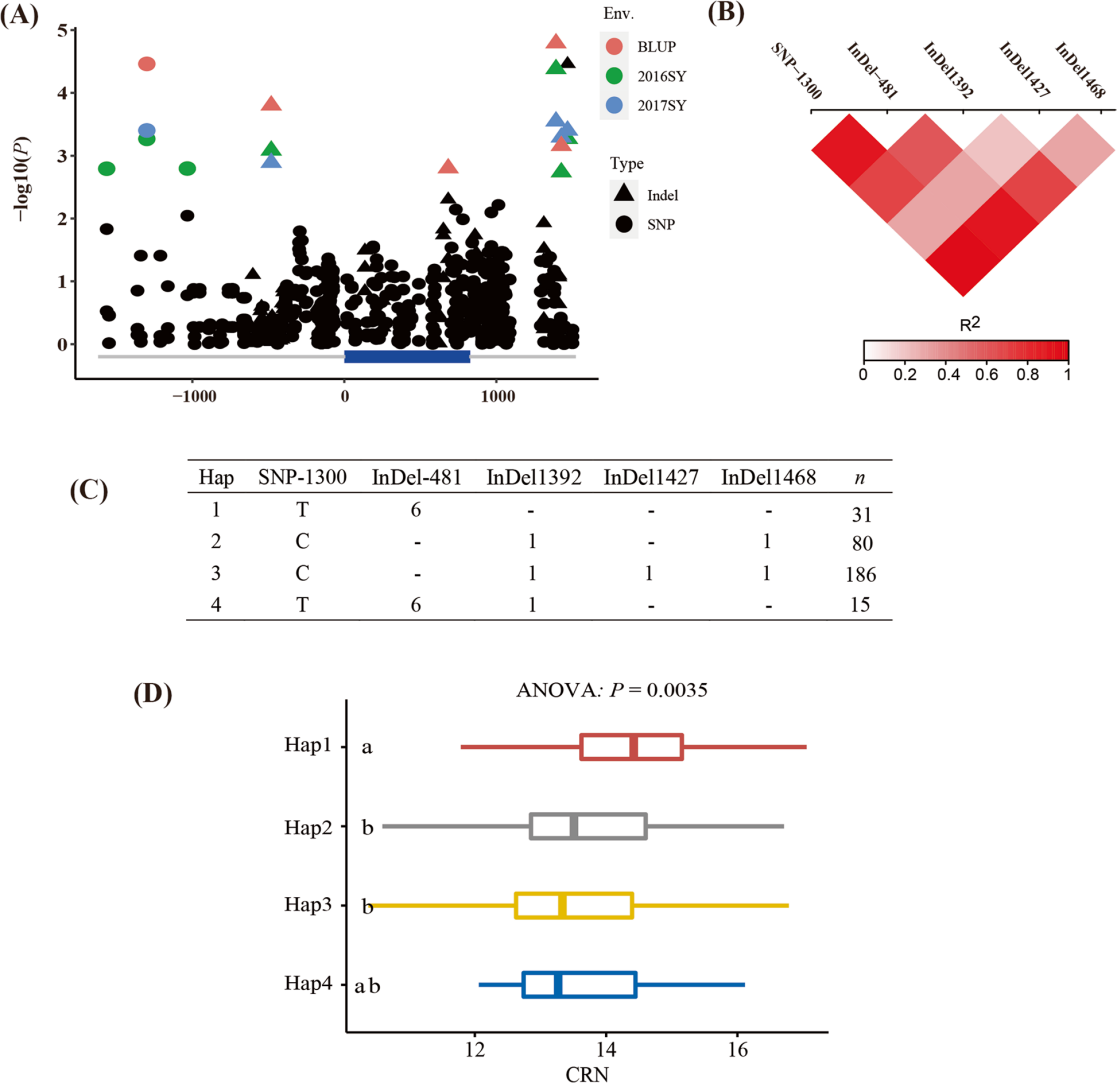
Candidate gene association analysis and haplotype analysis of GRMZM2G141205. (**A**) Manhattan plot for CRN in different environments. (**B**) pair-wise linkage disequilibrium (LD) of variants in GRMZM2G141205. (**C**) Haplotypes of GRMZM2G141205 among natural variations in inbred lines. (**D**) Phenotypic differences between different haplotypes. Levene's test (*P* = 0.93) indicated that the variance is homogeneous, so one-way ANOVA was used for comparisons between different haplotypes. The protected LSD post hoc test (*a* = 0.05) was used for multiple comparison tests. Different letters indicated that significant difference between haplotypes were observed (*P* < 0.05)

To investigate the associations between the allelic and phenotypic variations in the association panel, one gene was selected for resequencing. Our GWAS results identified SNP_6_146692801 (Table S2) as a significant SNP associated with CRN in 2016. The GRMZM2G141205 gene, encoding an AUX/IAA transcriptional regulator, was located 5.2 kb downstream of SNP_6_146692801. We analysed the GRMZM2G141205 genomic region comprising 3,128 base pairs (bp), including the 1,613-bp upstream region, the 828-bp coding region, and the 687-bp downstream region (after the translational termination site). A total of 243 sequence variations were identified (minor allele frequency [MAF] ≥ 0.05), including 202 SNPs and 41 InDels. A marker–trait association analysis based on the MLM detected four SNPs and five InDels significantly associated with CRN, including five variants (SNP-1300, InDel-481, InDel-1392, InDel-1427, and InDel-1468) that were detected in 16SY and 17SY as well as the BLUP values for the three environments (Table 4; Fig. 3A, B). On the basis of these five variants, four major haplotypes, which were present in more than 15 lines, were revealed in all tested inbred lines (Fig. 3C). Hap1, 2 3 and 4 including 31, 80, 186 and 15 lines, respectively. The ANOVA revealed that a significant phenotypic difference in the CRN was observed among the haplotypes, with Hap1 associated with the most crown roots (Fig. 3D).

**Table 4:** Significant markers associated with crown root number by GRMZM2G141205 based association analysis

Env	Marker	Alleles	-log10(*P*)	*R*^*2*^	Type	Mutation
2016SY	SNP-1563	C/G	2.94	0.035	upstream	
Mean	SNP-1300	C/T	4.14	0.050	upstream	
2017SY	SNP-1300	C/T	3.14	0.046	upstream	
2016SY	SNP-1300	C/T	2.89	0.034	upstream	
2016SY	SNP-1033	A/G	2.94	0.035	upstream	
Mean	InDel-481	–––/CGCCTA	3.76	0.045	upstream	
2017SY	InDel-481	–––/CGCCTA	2.82	0.040	upstream	
2016SY	InDel-481	–––/CGCCTA	2.98	0.035	upstream	
Mean	SNP189	C/T	3.01	0.037	exon	Stop gained
2016SY	InDel651	–––––––/AGACGGGGTCGCGG	2.76	0.036	exon	Frameshift
Mean	InDel683	––––––/CCTCCTGGTCGC	3.78	0.057	exon	Insert four amino acids
2016SY	InDel683	––––––/CCTCCTGGTCGC	3.52	0.058	exon	Insert four amino acids
Mean	InDel1392	T/-	5.37	0.068	downstream	
2017SY	InDel1392	T/-	3.30	0.048	downstream	
2016SY	InDel1392	T/-	3.75	0.047	downstream	
Mean	InDel1427	A/-	5.42	0.068	downstream	
2017SY	InDel1427	A/-	4.05	0.062	downstream	
2016SY	InDel1427	A/-	5.17	0.068	downstream	
Mean	InDel1468	A/-	4.14	0.050	downstream	
2017SY	InDel1468	A/-	3.14	0.046	downstream	
2016SY	InDel1468	A/-	2.89	0.034	downstream

## Materials and Methods

### Plant materials and phenotyping

The panel used in this study was composed 316 inbred lines which was mainly collected in China (Table S3), all the accessions were stock in Key Laboratory of Plant Functional Genomics of the Ministry of Education, Yangzhou, China [28]. The panel was mainly clustered into three groups referred to as Lancaster, Reid, and Tang SPT according to their known pedigrees and germplasm [29]. The panel were planted in three field trials in Sanya from 2015 to 2017. The field trial was conducted in a completely randomized block design of one-row plots with two replications. Each row was 3 m long, 0.5 cm wide and contained 11 plants. Shovelomics was conducted to evaluate three crown root traits at maturation stage [30]. Six healthy plants were randomly selected for excavation using standard shovels in each line. Shoot-borne roots formed in soil are designated crown roots, while shoot-borne roots initiated above soil level are termed brace roots. The crown root should emerge on the node situated flush with the soil surface. Here, the excavated plant was shaken briefly to remove most of the soil, the brace roots were first cut with a cutter knife, and the crown root on top layer could then be easily distinguished. The roots on top layer of crown root (first layer crown root) were selected to measure crown root angle (CRA), number (CRN), and diameter (CRD, mm) [19]. Root angles were measured with a protractor as degrees from horizontal: horizontal roots were classified as 0^◦^ and vertical roots as 90^◦^. Selected crown roots were cut off at 1 cm from the top of the root, and root diameter was measured with a vernier caliper. Each observation of phenotypic value was assumed following a linear model: *y*_ijk_ = *μ* + *G*_*i*_ + *E*_*j*_ + *GE*_*ij*_ + *B*_*k*_*(E*_*j*_*)* + *e*_*ilk*_, where *μ* represents the population mean, *G*_*i*_ represents the genotype effect, *E*_*j*_ represents the environment effect, *GE*_*ij*_ represents genotype-by-environment interaction, *B*_*k*_*(E*_*j*_*)* represents the block effect, and *e*_*ilk*_ represents the random error. The best unbiased linear predictive value (BLUP) of each trait was also calculated using a mixed linear model in R package “lme4”. The “lme4” package was used to estimate genotypic (${\sigma }_{g}^{2}$), G-E interaction (${\sigma }_{ge}^{2}$) and error variances (${\sigma }_{e}^{2}$). The broad-sense heritability ${h}^{2}={\sigma }_{g}^{2}/({\sigma }_{g}^{2}+{\sigma }_{ge}^{2}/e+{\sigma }_{e}^{2}/re)$, *e* and *r* represent the number of environments and blocks in each environment [31].

### Genotypic data and genome-wide association analysis

The panel of 316 inbred lines was genotyped using the genotyping-by-sequencing strategy [28]. After quality control (missing rate ≤ 20%; minor allele frequency ≥ 0.05) 140,421 SNPs remained for GWAS. The number of SNPs on each chromosome ranged from 10,344 (chromosome 9) to 19,513 (chromosome 1). Most SNPs (50.31%) were located outside of genic regions, of those in genic regions, 14.51% SNP were located within introns, which is more than that in exons (3.50%). The principal component analysis (PCA) was also performed with TASSEL, and the top five principal components were used to create a population structure matrix to control the population structure. Kinship matrices were calculated using the centred IBS method in TASSEL to estimate the genetic relatedness among individuals. A GWAS was conducted with two single-locus methods: GLM (with PC) and MLM (with PC and kinship) in TASSEL. FarmCPU [32], FASTmrEMMA [33] and FASTmrMLM [34] and were used for multi-locus GWAS. FarmCPU was implemented in the R package GAPIT3 with FarmCPU model (with PC). FASTmrEMMA and FASTmrMLM were conducted with the software package mrMLM.GUI V3.2 with five PCs and kinship to control structure. For GLM, MLM and FarmCPU, the *P* value threshold was 7.12 × 10^−6^ (1/*n*, where *n* is the number of SNPs). A suggestive LOD threshold value of 3.0 was used for FASTmrEMMA and FASTmrMLM [33, 34]. The LD decay was determined by software PopLDdecay [35], and an average LD across all chromosomes decayed to *r*^*2*^ = 0.20 in approximately 50 kb [36]. The significant SNPs were grouped into one region if the LD between the two neighboring SNPs was *r*^*2*^ > 0.6. The grouped region was detected in at least two environments or by at least two models was considered as a QTL, and SNPs with the minimum *P* value in a QTL were considered as the lead SNPs. The positions of the QTL identified in this study were compared the confidence interval of the QTL reported from previous biparental studies to identify overlapped QTL by different populations [19].

### Candidate gene analysis and gene-based association mapping

All the potential candidate genes within 50 kb of the detected loci were identified. The expression data and gene annotation information were collected from maizeGDB database (http://www.maizegdb.org) [37, 38]. The physical locations of the genes and SNPs were based on the maize B73 RefGen_V3 genome (version 5b.60).

Genomic DNA was extracted from the fresh young leaves of the 316 inbred lines. The candidate genes from the tested inbred lines were sequenced with the targeted sequence capture technology of the NimbleGen platform [39] by BGI Life Tech Co., Ltd. A multiple sequence alignment was analysed with the MAFFT software (v7.313). Gene-based polymorphisms were identified with TASSEL 5.2, with a minor allele frequency (MAF) ≥ 0.05. The significance of the association between maize SNPs and target traits was conducted with the MLM model (MLM + PCA + Kinship) in TASSEL 5.2. The *P* value threshold to control the genome-wide type 1 error rate was 1/*n* (where *n* is the number of markers for the candidate gene) [40]. To test the effect of unequal sample sizes between groups, Levene's test was used to test if different groups have equal variances. The result indicated that the variance is homogeneous (*P* = 0.93), so one-way ANOVA was used for comparisons between different haplotypes. The protected LSD post hoc test (*a* = 0.05) was used for multiple comparison tests.

## Discussion

Because the RSA is a key determinant of plant anchorage and the efficient uptake of nutrients and water, it directly influences crop yield potential [3, 12]. Breeding crops with an optimal RSA for capturing soil resources is a promising strategy for increasing yields under stress conditions [41]. However, the limited available information regarding the genetic mechanism underlying RSA development has been an obstacle for molecular marker-assisted breeding. Earlier research proved that CRN, CRA, and CRD are critical factors that define the maize RSA [11, 16, 42]. The challenges associated with evaluating root traits in the field have hindered the characterisation of the genetic mechanisms regulating root traits. Several new methods have recently been developed for relatively high-throughput and accurate analyses of roots under field conditions [43]. In this study, shovelomics-based experiments were conducted to evaluate the CRA, CRD, and CRN of 316 inbred lines at maturity in three field trials. Moreover, a GWAS was performed to elucidate the natural variations in the crown root traits.

Genome-wide association studies have identified genes responsible for complex plant traits [44]. Analyses involving MLM-based (mixed linear model) single-marker associations are commonly used to detect important loci related to complex plant traits, but their stringent significance thresholds resulting from large-scale multiple testing may prevent the identification of many critical loci [44]. And most quantitative traits are controlled by a few genes with large effects and numerous polygenes with minor effects. However, the current single-marker associations do not match the true genetic model for these traits [33]. In multi-locus GWAS, the Bonferroni correction is replaced by a less stringent selection criterion. Multi-locus approach such as FASTmrMLM and FASTmrEMMA shows high statistical power in QTN detection, model fit and low false positive rate [33, 45]. In this study, a GWAS was conducted using two single-locus methods and three multi-locus methods. A total of 174, 4, 61, 210, and 33 SNPs were identified with GLM, MLM, FASTmrEMMA, FASTmrMLM, and FarmCPU, respectively. Twenty-nine SNPs were identified by at least two methods, including SNP_5_158203765 and SNP_4_235330942, which were detected by four GWAS methods except MLM. We also detected 23 variants in at least two environments. After clumped, a total of 12, 10 and 16 QTL were detected for CRA, CRD and CRN. Linkage and GWAS analyses are two complementary approaches commonly used to clarify the genetic basis of complex traits. Additionally, these methods may be used for cross-validations [46]. We compared our GWAS results with the QTL identified in the recombinant inbred line population, which was included in an earlier evaluation of the same root traits in three field trails [19]. A total of three QTL were located in previous QTL intervals. Four candidate genes located in these QTL. Q6 was overlapped with QTL for seminal number, and two candidate genes were located in the confidence interval [26]. The consistency in the findings confirmed the accuracy of the GWAS results.

Auxin is a common regulator of plant root development. The auxin–TIR1/ABF2–AUX/IAA–ARF–LBD pathway is involved in post-embryonic root development in cereals and Arabidopsis [47]. The Aux/IAA proteins are key regulators of the nuclear auxin response pathway, and many of them can modulate root development in Arabidopsis, rice, and maize [48–50]. In maize, mutations to ROOTLESS WITH UNDETECTABLE MERISTEMS 1 (RUM1; an Aux/IAA protein) that prevent auxin-mediated degradation lead to lateral root defects [50]. Another Aux/IAA-related gene, rul1, is a duplicated homologue of rum1, which controls SR and LR initiation in maize [51]. In the current study, we identified an Aux/IAA protein-encoding genes, GRMZM2G141205, which were associated with CRN. The natural variations in GRMZM2G141205 were validated by resequencing and a haplotype analysis, and five variants were significantly associated with CRN. Here, two variants were in the upstream region of GRMZM2G141205. A collection of studies has shown that variations in the genes upstream may affect the gene expression and further lead to plant phenotypic alterations. For example, a CACTA-like transposon insertion in the upstream promoter region of ZmCCT10 in maize disrupts gene expression and attenuates photoperiod sensitivity under long-day environments [52]. An SNP in the promoter region of ZEA CENTRORADIALIS 8 (*ZCN8*) affected flowering time by altering the level of gene expression [53]. SNP-1300 and InDel-481 were the potential variants that may alter gene expression of GRMZM2G141205 to regulate CRN. It is worth noting that the promoter sequences in this study were 1613 bp, and some important variations maybe located more than 2000 bp from the start codon, such as a Hopscotch element was ~ 60 kb upstream of tb1 [54]. Cytokinin and auxin have antagonistic effects on root development [47]. O-glucosylation was common modification of cytokinins, O-glucosyltransferase OscZOG1 regulates root and shoot development and formation of agronomic traits in rice [55]. Over-expression of a zeatin O-glucosylation gene in maize leads to shoot growth retardation and increased root mass and branching [56]. In the current study, GRMZM2G175910 encoded a cytokinin-O-glucosyltransferase was associated with CRA. We also found that GRMZM2G138511 encoded a heat shock protein was associated with CRN. Previous study reported the role of heat shock protein 101 in the stimulation of nodal root development [57]. Whether these candidate genes affect maize crown root development will need to be experimentally verified (e.g., by targeted mutagenesis).

Genetic improvement of the root system architecture has been recognized as an important approach to enhance crop production. [10]. Marker-assisted selection and genomic selection are promising breeding strategy for improving complex traits. The identified significant SNP could be used to develop molecular markers to improve root traits, especially in stress conditions. Fewer crown root number enhances nitrogen acquisition from low-nitrogen soils in maize [10], so hap 2 and 3 of GRMZM2G141205 with fewer crown root could be useful to improve nitrogen use efficiency in maize. Further, natural variations of traits in maize appear to be predominantly controlled by regulatory variants[58], in present study, most variations in GRMZM2G141205 was in upstream and downstream regulatory regions. With revolutionary genome-editing technologies, promoter engineering as a promising solution, could produce desirable effects on traits of interest [58, 59]. The identified SNP may be potential target that can be used to alter the root system architecture.

In Conclusion, three crown root traits (CRA, CRD, and CRN) were evaluated in three field conditions in 316 maize inbred lines, a total of 38 QTL were identified by single-locus and three multi-locus GWAS. Combining the gene annotation information and the expression profiles, 3 genes including GRMZM2G141205 (IAA), GRMZM2G138511 (HSP) and GRMZM2G175910 (cytokinin-O-glucosyltransferase) were selected as potentially candidate genes related to crown root development. GRMZM2G141205, encoding an AUX/IAA transcriptional regulator, was resequenced in all tested lines. Five variants were identified significantly associated with CRN in different environments. These significant variants could be used to develop functional markers to improve root traits.

## Supplementary Information

**Additional file 1:** Supplementary **Table S1**. Significant SNPs associated with crown root traits detected by GWAS; Supplementary **Table S2**. The list of candidate genes located within the ± 50-kb genomic regions of the identified QTL. Supplementary **Table S3**. The list of 316 inbred lines used in this study.

**Additional file 2:** Supplementary **Figure S1** GWAS results for crown root angle by three GWAS methods in different environments. The grouped region that was detected in at least two environments or by at least two models was displayed in this figure. Supplementary **Figure S2** GWAS results for crown root diameter by three GWAS methods in different environments. The grouped region that was detected in at least two environments or by at least two models was displayed in this figure.

## Data Availability

All data analyzed during this study are included in the supplementary information files, and genotypic data have been deposited in the Sequence Read Archive (https://www.ncbi.nlm.nih.gov/bioproject/PRJNA683126 or https://trace.ncbi.nlm.nih.gov/Traces/study/?acc=SRP296905).
