# Supplementary material for: Exploiting natural variation in crown root traits via genome-wide association studies in maize
Source: BMC Plant Biol. 2021 Jul 23;21:346. doi: 10.1186/s12870-021-03127-x (PMC8299645; doi:10.1186/s12870-021-03127-x)
Supplement: Supplementary file 1 — Additional file 1: Supplementary Table S1. Significant SNPs associated with crown root traits detected by GWAS; Supplementary Table S2. The list of candidate genes located within the ± 50-kb genomic regions of the identified QTL. Supplementary Table S3. The list of 316 inbred lines used in this study. [file 12870_2021_3127_MOESM1_ESM.docx]

**Supplementary Figures**

Exploiting natural variation in crown root traits via genome-wide association studies in maize

Houmiao Wang^1^#, Xiao Tang^1^#, Xiaoyi Yang^2^, Yingying Fan^3^, Yang Xu^3^, Pengcheng Li^2^, Chenwu Xu^1^* and Zefeng Yang^1^*

^1^ Jiangsu Key Laboratory of Crop Genetics and Physiology/ Key Laboratory of Plant Functional Genomics of the Ministry of Education/ Jiangsu Key Laboratory of Crop Genomics and Molecular Breeding, Yangzhou 225009, China

^2^ Jiangsu Co-Innovation Center for Modern Production Technology of Grain Crops, Yangzhou University, Yangzhou 225009, China

^3^ Joint International Research Laboratory of Agriculture and Agri-Product Safety of Ministry of Education of China, Yangzhou University

***** Corresponding authors: [cwxu@yzu.edu.cn](mailto:cwxu@yzu.edu.cn); [zfyang@yzu.edu.cn](mailto:zfyang@yzu.edu.cn); Tel: 86-0514-87979358; Fax: 86-0514-879968

**Supplementary Figure. 1** GWAS results for crown root angle by three GWAS methods in different environments.

**Supplementary Figure. 2** GWAS results for crown root diameter by three GWAS methods in different environments.


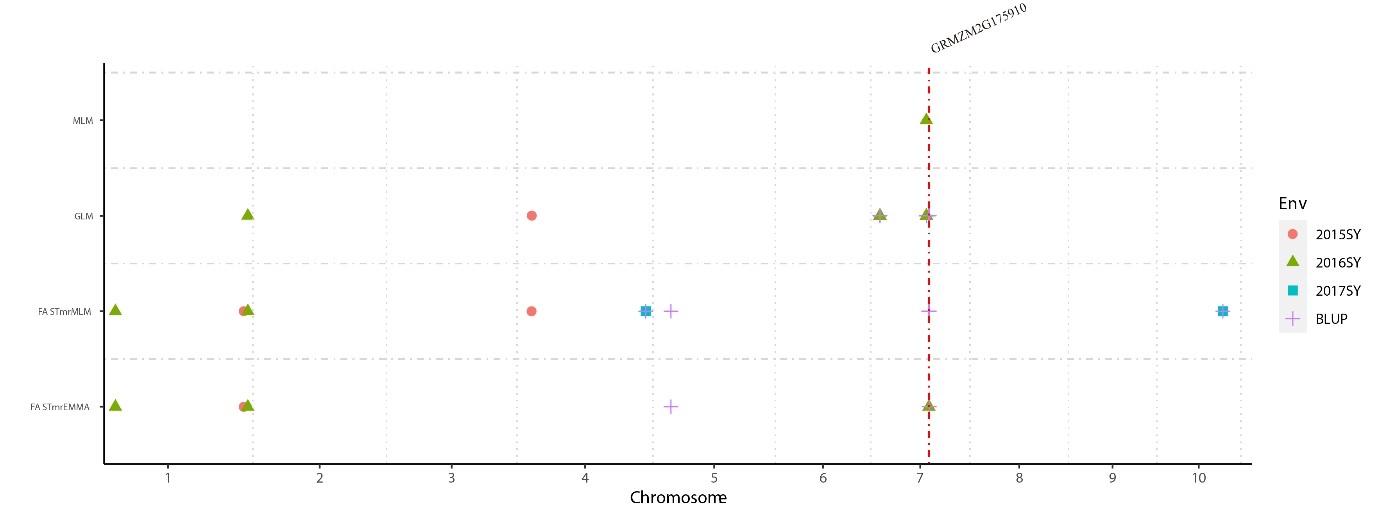


**Supplementary Figure. 1 GWAS results for crown root angle by three GWAS methods in different environments.** The grouped region that was detected in at least two environments or by at least two models was displayed in this figure.


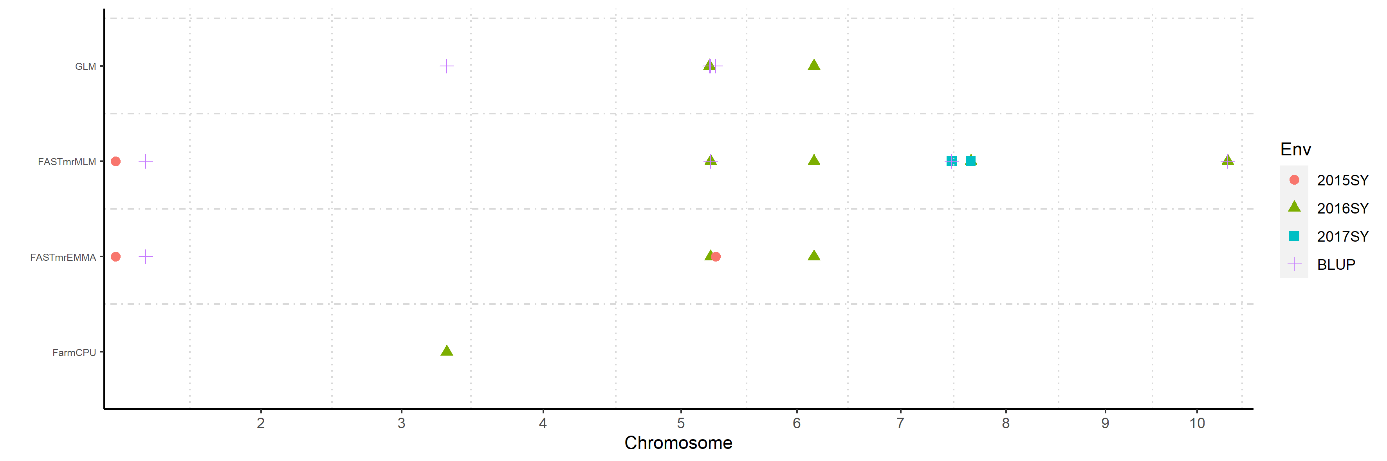


**Supplementary Figure. 2 GWAS results for crown root diameter by three GWAS methods in different environments.** The grouped region that was detected in at least two environments or by at least two models was displayed in this figure.
